# The impact of ovarian endometrioma and endometriotic cystectomy on anti-Müllerian hormone, and antral follicle count: a contemporary critical appraisal of systematic reviews

**DOI:** 10.3389/fendo.2024.1397279

**Published:** 2024-05-10

**Authors:** Johnny S. Younis, Hugh S. Taylor

**Affiliations:** ^1^ Reproductive Medicine, Department of Obstetrics and Gynecology, Tzafon Medical Center, Poriya, Israel; ^2^ Azrieili Faculty of Medicine in Galilee, Bar-Ilan University, Safed, Israel; ^3^ Department of Obstetrics, Gynecology and Reproductive Sciences, Yale School of Medicine, New Haven, CT, United States

**Keywords:** endometrioma, endometriotic cystectomy, ovarian reserve, Anti-Müllerian hormone, antral follicle count, meta-analysis

## Abstract

Currently, three crucial questions regarding the reliability of ovarian reserve measures in women with ovarian endometrioma during the reproductive age are being discussed. Firstly, the effects of endometriotic cystectomy on short and long-term ovarian reserve. Secondly, the accuracy of serum anti-Müllerian hormone (AMH) and antral follicle count (AFC) in estimating ovarian reserve in these cases. Thirdly, the impact of endometrioma itself on the ovarian reserve over time in such cases. The purpose of the present review is to critically assess available systematic reviews and meta-analyses that have explored these questions. Nine eligible reviews were found following a systematic search on PubMed.com and similarly assessed. These reviews varied considerably regarding the level of evidence, as per an identical comprehensive scoring system. Moderate to high-quality evidence demonstrates that endometriotic cystectomy, by the stripping technique, adversely affects ovarian reserve in the short and long term, up to 9-18 months post-surgery. Damage to ovarian reserve was considerable but more pronounced in bilateral cases than unilateral cases, equivalent to 39.5% and 57.0%, respectively. Repeat endometriotic cystectomy is detrimental to ovarian reserve. The impact of endometrioma diameter on ovarian reserve before or after surgery is still unclear. Moderate to high-quality evidence, relying on simultaneous assessment of both ovarian reserve measures, shows that AMH is sensitive while AFC is not in cases undergoing ovarian cystectomy. AMH should be the biomarker of choice for counseling and managing women with endometrioma in their reproductive age, especially before surgery. While there is some evidence to show that endometrioma per se may harm ovarian reserve, this evidence is not robust, and there is good-quality evidence to challenge this notion. It is necessary to conduct further targeted RCTs, systematic reviews, and meta-analyses based on solid methodological grounds to increase the level of evidence, refine quantitative estimates, investigate open questions, and decrease heterogeneity.

## Introduction

Ovarian reserve appraisal in women with ovarian endometrioma continues to be a challenging and debated topic in reproductive medicine. This diagnostic complexity arises from several fundamental inquiries about disease pathogenesis and patient management. These include the potential influence of endometrioma per se on ovarian reserve, the adverse effect of ovarian surgery on ovarian reserve, and the adequacy of the most established ovarian reserve measures, anti-Müllerian hormone (AMH) and antral follicle count (AFC) to accurately assess ovarian reserve in these women.

While standards for evidence-based clinical guidelines stipulate the use of systematic reviews, uncritically accepting the results of a single systematic review may have several masked risks (1). Indeed, several systematic reviews and meta-analyses exploring ovarian reserve appraisal in cases with ovarian endometrioma were published in the last decade. However, they had contrasting results, instigating confusion and potentially leading to obverse clinical management.

In the general population of reproductive age, among various tests, AFC and AMH are considered the most sensitive and reliable non-invasive methods of ovarian reserve evaluation (2, 3), with no superiority dispute between the two. Two independent systematic reviews and meta-analyses examined the impact of endometriotic cystectomy during the reproductive age, the first employing AMH and the second AFC, and they have reached contradictory conclusions. The first, by Raffi et al. in 2012, reported that surgery had a harmful effect on AMH (4), while the second, by Muzii et al. in 2014, using AFC, found that it did not affect the ovarian reserve (5). Drawing an inference on whether endometriotic cystectomy genuinely influences the ovarian reserve from these two independent systematic reviews is limited. This controversy may have resulted from different methodologies and standardizations of each systematic review containing the surgical technique, endometrioma size, laterality, previous ovarian surgery, and postoperative time interval evaluations. This controversy may also raise questions about the suitability and reliability of both AMH and AFC as ovarian reserve measures in women with endometrioma.

The purpose of the present review is to critically assess available systematic reviews and meta-analyses that have explored the impact of ovarian endometrioma and endometriotic cystectomies on ovarian reserve measures, AMH, and AFC. Looking deeper into the methodology of these systematic reviews may clarify the bias range, uncover the discrepancies between the reports, distinguish high-quality assessments, explain clinical implications, and assist in decision-making.

## Materials and methods

To reach the objective of this review, the PubMed database was searched for manuscripts that included the syntaxes (and their MeSH terms): endometriosis OR endometrioma AND ovarian reserve OR anti-Müllerian hormone OR antral follicle count. The research was restricted to systematic reviews (with or without meta-analysis) published in peer-reviewed journals for clinical studies performed in humans and English from inception until November 2, 2023. Systematic reviews that examined ovarian reserve measures, specifically AMH and AFC, in women with ovarian endometrioma before or after surgery were applicable for evaluation.

Systematic reviews that targeted women with non-endometriotic ovarian cysts, gonadal or non-gonadal malignancy, polycystic ovary syndrome, diabetes mellitus, thyroid disease, human immunodeficiency virus, autoimmune disease (such as systemic lupus erythematosus or rheumatoid arthritis), inflammatory bowel disease, and BRCA variants were excluded. Furthermore, systematic reviews that targeted females in childhood, adolescence, and peri- or postmenopausal were omitted from the evaluation. In addition, papers that evaluated women following a minimally invasive approach to managing an endometrioma, such as ablation, laser vaporization, or ethanol sclerotherapy, were excluded. Additionally, papers that aimed to evaluate salpingectomy, hysterectomy, and uterine artery embolization were omitted from the evaluation. As well, reviews that targeted acupuncture in women with endometriosis or explored the hemostatic approach following endometriotic cystectomy were excluded.

## Results

### Included and excluded studies

One-hundred fifty-seven systematic reviews were identified from the PubMed database. One-hundred and thirty-one were excluded following title and abstract reading. The remaining 26 systematic reviews were assessed for eligibility following a full manuscript inspection. Eight publications were excluded since they targeted minimally invasive methods of endometrioma management (laser and ablation) (6–8) or different hemostatic methods during conventional endometriotic cystectomy (bipolar, suture) (9–13). Another five publications targeted IVF outcomes, excessive response, or livebirth were excluded (14–18). In addition, four publications did not exclusively target women with endometrioma and were omitted from the evaluation (19–22).

Nine systematic reviews were eligible for critical evaluation and are summarized in **Table 1** (4, 5, 23–29). According to the Journal Citation Reports 2022, all reviews except one (28) were published in a Journal with a well-established impact factor. Furthermore, all but one review (23) conducted a meta-analysis of the accumulated data, reaching a quantitative evaluation. In addition, eight of the nine systematic reviews targeted ovarian endometrioma, while one targeted women with endometriosis (27). Seven reviews targeted women undergoing endometriotic cystectomy (4, 5, 23, 25, 26, 28, 29), and two focused on women with endometrioma or endometriosis before surgery (24, 27). The primary outcome measure was AMH in six reviews and AFC in two (5, 27). Only one review targeted both AMH and AFC at the same time in parallel as a primary outcome measure (29).

**Table 1:** Summary of systematic reviews exploring ovarian reserve measures (AMH and AFC) in cases with ovarian endometrioma.

Authors	Journal	Primary outcome measure	Number of studies	Number of women	Meta-analysis performed	Studies included Prospective or RCT or retrospective NRSI#		Risk of bias evaluation
Raffi et al., 2012 (4)	J Clin Endocrinol Metab	Endometriotic cystectomy impact on AMH	8	237	yes	All prospective	combined	Newcastle-Ottawa scale
Somigliana et al., 2012 (23)	Fertil Steril	Endometriotic cystectomy impact on AMH	11	344	no	combined	combined	No
Muzii et al., 2014 (5)	Human Reprod	Endometriotic cystectomy impact on AFC	13	597	yes	combined	combined	No
Muzii et al., 2018 (24)	Fertil Steril	Endometrioma impact on AMH	17	968	yes	combined	combined	Newcastle-Ottawa Scale
Younis et al., 2019 (25)	Hum Reprod Update	Unilateral and bilateral endometriotic cystectomy impact on AMH	12	783	yes	All prospective	combined	Newcastle-Ottawa scale
Nankali et al., 2020 (26)	Health and Quality of Life Outcomes	Unilateral and bilateral endometriotic cystectomy impact on AMH	19	1825	yes	combined	combined	CONSORT checklist
Tian et al., 2021 (27)	RBMOnline	Endometriosis impact on AFC	15	888	yes	combined	combined	Newcastle–Ottawa Scale and others
Moreno-Sepulveda et al., 2022 (28)	JBRA Assisted Reproduction	Endometriotic cystectomy impact on AMH	36	4374	yes	combined	combined	Newcastle–Ottawa Scale
Younis et al., 2022 (29)	Am J Obstet Gynecol	Endometriotic cystectomy impact on parallel AFC and AMH evaluations	14	650	yes	All prospective	combined	Newcastle-Ottawa scale

#RCT, randomized controlled studies; NRSI, non-randomized studies of intervention.

### Critical assessment of included systematic reviews

To critically and similarly assess in a transparent approach the preparation, conduct, and rating of the certainty of the evidence of the eligible systematic reviews, an *a priori* list of key entries was formulated. This comprehensive list included twenty fundamental entries applicable to the research question, the literature search methodology, the handling of included and excluded studies, and the eligibility of studies for being adequate for meta-analysis. In addition, significant points relevant to ovarian endometrioma and their impact on both ovarian reserve tests, AMH and AFC, were incorporated, including previous ovarian surgery, endometrioma diameter, and laterality. Furthermore, the design and risk of bias evaluation of studies found eligible for meta-analysis in each review were addressed. The timing and methodology of the ovarian reserve tests were also examined. Moreover, the methodology of the meta-analysis and the efforts invested to explain estimates with high heterogeneity were explored. Finally, the funding sources and conflict of interest in conducting the systematic review were assessed.

Since all meta-analyses analyzed a combination of randomized and non-randomized studies, a modification of the A Measurement Tool to Assess Systematic Reviews (AMSTAR-2) tool was incorporated into the list of crucial entries (1). Significant issues pertinent to the subject of ovarian endometrioma were also added to this list. **Table 2** summarizes the performance of all nine systematic reviews found suitable in the search. Each entry in the list received a score of 2, 1, and 0, depending on whether it was implemented (and documented), partially implemented, or not in each specific review. As shown in **Table 2**, a wide variation between the total scores was found among the nine systematic reviews, indicating a diversity in the quality of evidence.

**Table 2:** Critical appraisal of the systematic reviews exploring the impact of endometrioma and endometriotic cystectomy on ovarian reserve measures.

	Raffi et al., 2012 (4)	Somigliana et al., 2012 (23)	Muzzi et al., 2014 (5)	Muzzi et al., 2018 (24)	Younis et al., 2019 (25)	Nankali et al., 2020 (26)	Tian et al., 2021 (27)	Moreno-Sepulveda et al., 2022 (28)	Younis et al., 2022 (29)
The research question and primary outcome measure are clearly stated and clarified	2	2	2	2	2	2	2	2	2
The study protocol and methodology are well-explained	2	2	2	2	2	1	2	1	2
The protocol is a priori registered on an open-access online database of systematic reviews	0	0	0	0	2	0	0	0	2
The literature search strategy is comprehensive	2	1	2	2	2	1	2	2	2
The study selection is performed in duplicates	1	2	2	2	2	0	2	2	2
The data extraction is performed in duplicates	2	2	2	2	2	0	2	1	2
The excluded studies are clearly described and justified	2	2	2	2	2	1	2	0	2
The included studies are adequately detailed	2	2	2	2	2	1	2	1	2
Cases of previous endometriotic cystectomy are excluded from the evaluation	1	2	2	2	2	0	0	0	2
The laterality of the endometrioma is taken into account	2	2	1	1	2	0	1	2	1
Endometrioma diameter is taken into account	2	1	1	1	1	0	0	1	1
All studies included are prospective in design	2	1	1	1	2	1	1	1	2
All studies included originated in randomized controlled studies	1	1	1	1	1	1	0	1	1
Postoperative ovarian reserve biomarkers were analyzed at standardized time points	1	2	1	NR	2	2	NR	2	2
The methodology of AMH and AFC is described	2	2	0	0	2	0	2	0	2
A satisfactory risk of bias evaluation of included studies is performed	2	0	0	2	2	1	2	2	2
Appropriate meta-analytic methods are performed to reach calculated estimates	2	NR	2	2	2	2	2	2	2
The high heterogeneity of estimates is satisfactorily explained	2	NR	1	0	2	0	2	0	2
The source of funding is avowed.	0	0	2	0	2	2	2	0	2
Potential sources of conflict of interest are declared	2	2	2	2	2	2	0	2	2
Total score	32	26	28	26	38	17	24	22	37

Each entry scoring: 2 – yes, 1 – partially yes, 0 – no.

NR, not relevant.

### Main findings of the systematic reviews

**Table 3** summarizes the primary findings of the nine systematic reviews included in this appraisal. In-toto, these reviews targeted two primary questions (outcome measures) employing the best available ovarian reserve tests in reproductive age, AMH, and AFC. The first is the impact of endometriotic cystectomy, and the second is the impact of endometrioma per se on ovarian reserve biomarkers. The contradictory results of the first three systematic reviews (4, 5, 23) dealing with the impact of endometriotic cystectomy have raised a third question (outcome measure) of which of the best available ovarian reserve tests, AMH and AFC, is more reliable in this setting.

**Table 3:** Summary of the primary findings of the systematic reviews examining the reliability of AMH and AFC in evaluating ovarian reserve in cases with endometrioma.

Study	Key conclusions
Raffi et al., (4)	Endometriotic cystectomy causes significant damage to ovarian reserve with up to 40% fall in serum AMH concentration.
Somigliana et al., (23)	Serum AMH is reduced after surgical excision of endometriomas, which supports surgery-related damage to ovarian reserve.
Muzii et al., (5)	AFC did not change after endometriotic cystectomy. The affected ovary had lower AFC both before and after surgery.
Muzii et al., (24)	Ovarian reserve evaluated with serum AMH is reduced in patients with ovarian endometriomas compared to both patients with other benign ovarian cysts and patients with healthy ovaries.
Younis et al., (25)	Endometrioma cystectomy is implicated in a considerable decrease in ovarian reserve. After 9-12 months, unilateral and bilateral cystectomy showed a significant and sustained serum AMH drop of 39.5% and 57.0%, respectively. Pre-operative serum AMH weighted mean difference did not differ between unilateral and bilateral groups, challenging the concept that endometrioma per se adversely affects ovarian reserve.
Nankali et al., (26)	Unilateral and bilateral endometriotic cystectomy decreases serum AMH levels significantly. Bilateral cystectomy decreases AMH levels more than unilateral surgery, and this reduction intensifies after six months.
Tian et al., (27)	Endometriosis is associated with reduced AFC and AMH, suggesting a reduction in ovarian reserve, especially in those with ovarian endometrioma and advanced stage.
Moreno-Sepulveda et al., (28)	Endometrioma surgery deleteriously affects short-, medium-, and long-term postoperative serum AMH levels. Bilateral endometriomas and endometriomas above 7 cm have been associated with a more significant decrease in AMH.
Younis et al., (29)	Endometriotic cystectomies are associated with a significant reduction in serum AMH levels but not in the AFC, and the detrimental effects on AMH are consistently detectable at the early, intermediate, and late postoperative time points. In women with endometrioma, AMH provides a more accurate assessment of the risk for iatrogenic depletion of the ovarian reserve.

The first two published systematic reviews exploring the impact of endometriotic cystectomy employing AMH showed decreased ovarian reserve following surgery (4, 23). However, a third systematic review in a similar setting employing AFC showed reassuring results with no change in ovarian reserve (5). As such, AFC in this review suggested a more specific biomarker of ovarian reserve in this setting since it controls for the laterality of the possible damage (5).

Our group corroborated the findings of Raffi et al. (4) and Somigliana et al. (23), employing AMH as an ovarian reserve biomarker (25). Furthermore, we showed that impairment of the ovarian reserve is sustained for 9-12 months postoperatively, which is more detrimental in bilateral than unilateral cases, consistent with 39.5% and 57.0%, respectively, from baseline. This was also verified by two later published systematic reviews (26, 28).

To resolve the discrepancy between AMH and AFC performance in cases with endometrioma, our group conducted an additional systematic review and meta-analysis. In this study, both AMH and AFC were targeted for each woman concurrently (overcoming unmeasured confounders), in the same setting (overcoming surgical technique differences), and at the same three postoperative time points, namely early (1-6 weeks), intermediate (2-6 months) and late (9-18 months), to overcome time-sensitive changes (25). In this review, endometriotic cystectomies were associated with a significant reduction in serum AMH levels but not in the AFC, with the detrimental effects on AMH consistently detectable at all three time points extending to 9-18 months postoperatively. These findings clearly showed that AMH is a more sensitive biomarker of damage to the ovary than AFC and should be recommended as the biomarker of choice for women with endometrioma counseling.

The main controversial and unsettled topic that is still ongoing today is whether endometrioma per se affects ovarian reserve. In the reviews by Muzii et al., 2018 (24) and Tian et al., 2021 (27), which examined the impact of endometrioma on ovarian reserve, a decrease in AMH and AFC levels was detected compared to controls, suggesting a negative impact on ovarian reserve. However, it is essential to note that while the first review targeted women with endometrioma, the second targeted women with endometriosis. Furthermore, both reviews pooled retrospective and prospective studies together. Interestingly, previous surgery was not cited as an exclusion criterion of eligible studies in the second review (24). In addition, neither review assessed the impact on the ovarian reserve over time. A detailed methodological assessment of these two systematic reviews is summarized in **Table 2**.

Conversely, in a previous systematic review, pooling only prospective studies and targeting endometriotic cystectomy in unilateral versus bilateral cases in the same setting, preoperative serum AMH levels did not significantly differ between the groups (25). If endometrioma per se had a deleterious effect on the ovarian reserve, cases with bilateral compared to unilateral endometrioma should have had lower serum AMH levels at baseline before surgery. These results challenge the concept that endometrioma per se adversely affects ovarian reserve, calling for further assessment and research to gain a deeper insight into this topic.

## Discussion

This review is a comprehensive and critical summary of all systematic reviews that assess the performance of AMH and AFC in managing cases of endometrioma. Nine eligible reviews were analyzed after conducting a systematic search on PubMed.com. The systematic reviews varied in terms of the level of evidence, as per an identical broad scoring system. Each review was evaluated transparently, using a pre-formulated list of 20 key entries essential to the systematic review’s methodology and including hefty aspects of ovarian endometrioma characteristics related to ovarian reserve measures. This methodology helps to assess the certainty of evidence in systematic reviews, strengthens clinical practice recommendations, and assists in decision-making.

Three fundamental questions linked to the reliability of ovarian reserve measures when tackling ovarian endometrioma in the reproductive age are in discussion nowadays. The first is the impact of endometriotic cystectomy on the short and long-term of ovarian reserve. The second is the reliability of AMH and AFC in accurately estimating ovarian reserve in cases with ovarian endometrioma. The third is the impact of endometrioma per se on ovarian reserve as a function of time in such cases.

Moderate to high-quality evidence shows that endometriotic cystectomy, which is typically performed using the striping technique, is harmful to the ovarian reserve. Following the operation, serum AMH levels significantly decrease, starting in the early postoperative period and continuing until the late postoperative period, which lasts 9-18 months (29). This negative effect is more pronounced in bilateral cases than unilateral cases, equivalent to an ovarian reserve decline of 39.5% and 57.0%, respectively (25). Consequently, this decrease could have a detrimental effect on the reproductive life span. Histological studies of endometriotic cystectomy back up this conclusion and indicate that this damage is due to the unintentional removal of normal ovarian follicles near the pseudo-capsule, which is almost unavoidable even with experienced surgeons (30–32). This iatrogenic damage seems to be a composite result of the manipulation of the cortex, tearing of tissue planes, bleeding, and coagulation injury.

Furthermore, there is moderate to high-quality evidence that AMH is a much more sensitive biomarker of ovarian reserve than AFC in cases with ovarian endometrioma. Therefore, it should be the ovarian reserve biomarker of choice for women’s guidance in this setting, especially before surgery. In such cases, counseling women relying merely on AFC may be misleading for clinical practice recommendations.

The results obtained in the recent meta-analysis, assessing both AMH and AFC in parallel, in the same women, in the same setting, and at the same postoperative time points (29), corroborate basically the conclusions of the earlier systematic reviews evaluating AMH or AFC separately (4, 5, 23). It is, therefore, clear now that the discordancy between these systematic reviews on ovarian reserve findings is not the result of variances in methodology and standardization but rather the low sensitivity of AFC in detecting ovarian reserve changes in cases with ovarian endometrioma. These findings also support the notion that AFC is underestimated in cases with an intact endometrioma during the reproductive age.

Four essential and relevant concerns to endometrioma should be considered when evaluating the reliability of ovarian reserve measures in this setting during reproductive age. These concerns include previous ovarian surgery, the diameter of the endometrioma, its laterality, and the postoperative timing of ovarian reserve evaluation. All of these concerns were incorporated into the list of 20 key entries formulated *a priori* to examine the level of evidence of each of the systematic reviews in this critical contemporary review.

It has been found that both recurrent endometriotic cystectomy and bilateral endometriotic cystectomy can have a severe damaging impact on ovarian reserve. Previous studies have shown that there is a significant risk of premature ovarian insufficiency, either early or late, in such situations (33–35). Furthermore, it is essential to consider the timing of ovarian reserve appraisal following surgery, as natural changes can occur over time. For example, in women in their 30s, the natural decline in AMH is about 5% per year (36). However, the impact of surgery can exceed this natural decline by a considerable margin equivalent to 5-10 years (25). In addition, the diameter of the endometrioma may also significantly affect the damage to the ovarian reserve caused by endometriotic cystectomy. Recent research suggests that the threshold to distinguish between endometriomas that may or may not interfere with the ovarian response is 4 cm in diameter (37). Conversely, other investigators have reported that in women with no previous history of ovarian surgery, serum AMH levels were increased in cases with large endometriomas (38, 39). Due to the significance of AMH level association with endometrioma diameter and its related broader implications, prospective well-conducted studies with repeat measures of endometrioma diameter and AMH concentrations are vitally required.

A third fundamental question, which is still debated and not yet settled, is whether endometrioma per se may adversely affect ovarian reserve in the reproductive age. In the *in-vitro* setting, endometrioma was shown to instigate intra-ovarian inflammatory reactions attributed to the high concentration of free iron, reactive oxygen species, proteolytic enzymes, and inflammatory molecules. Macrophages, cytokines, and vasoactive substances mediate these reactions, which may damage adjacent ovarian cortical tissue. Various molecular, histological, and morphological evidence supports such mechanisms. However, the evidence is far from being conclusive (40).

In the clinical setting, two previous systematic reviews have explored this question, the first employing AMH and the second AFC, showing that an intact endometrioma may harm ovarian reserve (24, 27). However, several methodological concerns were raised in the results section and **Table 2**. Furthermore, no changes were found in serum AMH levels at baseline comparing unilateral and bilateral endometrioma cases, challenging the concept that endometrioma per se might have a negative impact on ovarian reserve (29). Further targeted randomized, well-controlled studies and adequately designed systematic reviews and meta-analyses are needed to explore this question adequately.

We acknowledge that our search for systematic reviews relied on one search engine, PubMed.com. For common clinical questions, PubMed.com is a reliable engine that can narrow the search and provides the highest-quality, most relevant, and most readable hits. Cochrane, considered the gold standard for clinical systematic reviews, recommends searching PubMed.com as one of the most commended databases.

## Conclusions

Moderate to high-quality evidence shows that endometriotic cystectomy is detrimental to ovarian reserve. Ovarian reserve damage is more pronounced in bilateral than unilateral cases and in women with previous ovarian surgery. Furthermore, moderate to high-quality evidence demonstrates that AMH is significantly sensitive to surgical impact on ovarian reserve, while AFC is not. Serum AMH should be the ovarian reserve biomarker for counseling women with endometrioma during the reproductive age, especially before surgery. Although there is some evidence that endometrioma per se may harm ovarian reserve, it is not robust. Furthermore, there is good evidence to challenge this notion. The impact of endometrioma diameter on ovarian reserve before or following surgery is still unclear. Further targeted RCTs, systematic reviews, and meta-analyses based on solid methodological grounds are essential to investigate open questions, increase the level of evidence, refine quantitative estimates, and decrease heterogeneity.

## Author contributions

JY: Conceptualization, Data curation, Investigation, Methodology, Writing – original draft. HT: Supervision, Validation, Writing – review & editing.
